# Complete Genome Sequence of a Phycodnavirus, *Heterosigma Akashiwo* Virus Strain 53

**DOI:** 10.1128/genomeA.01279-16

**Published:** 2016-11-10

**Authors:** Yoshitoshi Ogura, Tetsuya Hayashi, Shoko Ueki

**Affiliations:** a Department of Bacteriology, Faculty of Medical Sciences, Kyushu University, Fukuoka, Japan; b Institute of Plant Science and Resources, Okayama University, Okayama, Japan

## Abstract

We report the complete genome sequence of *Heterosigma akashiwo* virus strain 53. The virus is a member of the *Phycodnaviridae*, one of the families regarded as giant double-stranded DNA viruses. The 274,793-bp genome contained 246 protein-coding and 3 tRNA-coding sequences.

## GENOME ANNOUNCEMENT

*Heterosigma Akashiwo* is a photosynthetic eukaryotic unicellular alga that belongs to the class Raphidophyceae*.* It is one of the bloom-causing algae, which is widely observed in Pacific Rim regions, including North and South America, eastern Asia, Oceania, and the Northern Atlantic region (1–12). *H. akashiwo* bloom is known to be terminated by algicidal bacteria (13–21) and viruses (22–24). *Heterosigma akashiwo* virus (HaV) was identified as one such bloom-terminating factor (24, 25). Its genome was characterized to be a linear double-stranded DNA (dsDNA), with an estimated size of ~290 kbp (25). It is a member of the *Phycodnaviridae*, one of the viral families regarded as giant dsDNA viruses that possess genomes larger than several hundred kilobase pairs in size (26).

Here, we report the complete genome sequence of *HaV* strain 53, originally isolated from the Itsukaichi Fishing Port in Hiroshima Bay, Japan (27). *HaV53* was propagated on *H. akashiwo*, and viral particles were collected by adding polyethylene glycol 8000 at a final concentration of 6% to the culture medium containing lysed hosts, followed by centrifugation of the mixture at 21,000 × *g*. The *HaV53* DNA was extracted from the purified HaV 53 particles by proteinase K digestion, followed by chloroform-isoamyl alcohol treatment and ethanol precipitation. A genomic DNA library was prepared using a Nextera XT DNA sample prep kit (Illumina), and 24 million reads were generated by HiSeq 2500 using the 100-bp paired-end mode. Reads with high-quality scores (>28) were assembled using *Platanus* (28), yielding five high-sequence-coverage contigs (73.2, 58.5, 57.6, 41.2, and 33.2 kb) derived from *HaV* and numerous low-coverage contigs derived from the host DNA. Gaps between the contigs were filled by sequencing of gap-spanning PCR products using ABI3130xl and Illumina MiSeq sequencers. The accuracy of assembly was confirmed by mapping the paired-end reads to the final assemblage using BWA (29).

The genome of *HaV53* was 274,792 bp in size, and the A+T content was 69.6%. It was predicted to contain 247 open reading frames (ORFs) by GeneMarkS (30) and 3 tRNAs by tRNAscan-SE (31). Among the 246 ORFs, 105 had significant hits in the NCBI nonredundant protein database (BlastX, searched with *E* value <10^-5^); 4 had best hits to the sequences previously reported for *HaV* strain 01 (accession numbers BAE06835.1, BAE06251.1, BAB69884.1, and BAB69883.1) and 23 has best hits to other *Phycodnaviridae* members. The 105 ORFs coded for polypeptides with a variety of functions, including gene regulation, metabolism, signal transduction, and ubiquitin-related protein regulation. As the sequence of *HaV53* reported here is the first complete genome sequence of *HaV*, it would help advance research on *Phycodnaviridae*.

### Accession number(s).

The annotated genome sequence of *HaV53* has been deposited in DDBJ/EMBL/GenBank under the accession number KX008963.
